# Performance test of Zn-astaxanthin complex-sensitized solar cell: effect of light intensity on open-circuit voltage and short-circuit current values

**DOI:** 10.55730/1300-0527.3653

**Published:** 2024-01-05

**Authors:** Septiani SEPTIANI, Winda RAHMALIA, Thamrin USMAN

**Affiliations:** Department of Chemistry, Faculty of Mathematics and Natural Science, Tanjungpura University, Pontianak, Indonesia

**Keywords:** Astaxanthin, complex, sensitizer, solar cell, stability

## Abstract

The sensitizer is one of the most essential dye-sensitized solar cell (DSSC) components. In the present research, a Zn-astaxanthin complex was investigated as a sensitizer, compared to pure astaxanthin. The complex with a 1:1 mole ratio between astaxanthin and Zn^2+^ was synthesized in a reflux reactor at 37–60 °C. The product was analyzed using Proton Nuclear Resonance (^1^H-NMR), which indicates the presence of chelate formation between Zn^2+^ with two atoms of oxygen on the terminal cyclohexane ring of astaxanthin. The interaction of sensitizers (astaxanthin and Zn-astaxanthin) on the photoelectrode surface in this study was analyzed using a Fourier Transform Infra-Red (FTIR) and Ultraviolet-Visible Diffuse Reflectance Spectroscopy (UV-Vis DRS). The FTIR spectra of photoelectrode immersed in Zn-astaxanthin show peaks of C=O stretching and vibration -OH group at 1730 and 1273 cm^−1^, respectively, and H-C-H stretching vibration with high intensity in 2939, 2923, and 2853 cm^−1^. The UV-Vis DRS analysis shows the band gap of photoelectrode (PE), photoelectrode immersed in astaxanthin (PE/astaxanthin), and Zn-astaxanthin (PE/Zn-astaxanthin) are 3.19, 1.65, and 1.59 eV, respectively. Under illumination intensity of 300 W/m^2^, the maximum energy conversion efficiency of DSSC with Zn-astaxanthin as sensitizer is (0.03 ± 0.0022)%, higher than DSSC with astaxanthin as sensitizer ((0.12 ± 0.0052)%). Up to 70 h of illumination, DSSC with Zn-astaxanthin as a sensitizer also has better stability than astaxanthin-based DSSC.

## 1. Introduction

Dye-sensitized solar cells (DSSCs) are clean and renewable alternative energy sources because they can convert solar energy into electricity without emitting CO_2_ gas. One component that affects the performance of the DSSCs is the sensitizer, which harvests light from the visible range to the infrared region [1]. Most of the dyes that have been used as sensitizers in DSSCs are Ruthenium complex (N3, N719, N945) synthetic dyes [2]. However, toxicity problems, scarce availability, and high cost caused by synthetic dyes have triggered intense research on natural dyes. It encourages researchers to switch to the used natural dyes, with cheaper raw material prices, abundant availability, environmentally friendly, biocompatible, and biodegradable easily [3]. Carotenoids have the potential to be applied as sensitizers for several reasons. First, carotenoids in nature serve as light-harvesting agents in the process of photosynthesis to produce energy, and they can absorb energies from the solar spectrum with a wavelength range of 380–550 nm and a high molar absorption of ~1 × 10^5^. Second, an electronic transition of the carotenoids occurs from the ground state (S_0_) to the second excited state (S_2_), which causes stronger absorption of light. This internal conversion from the excited state occurs to the lowest excited state (S_1_) and then to the ground state. Third, on light absorption, excited electrons from HOMO (Highest Occupied Molecular Orbital) to LUMO (Lowest Unoccupied Molecular Orbital) will be collected into the external circuit resulting in the flow of electric current. Furthermore, in the DSSC system, these electrons will be injected into the photoelectrode (TiO_2_ semiconductor) [4].

The use of carotenoid dyes as sensitizers in DSSC systems has been reported by several studies, as with bixin, *β*-carotene, norbixin, zeaxanthin, and astaxanthin resulting in energy conversion efficiencies of 0.08, 0.076, 0.13, 0.17, and 0.036%, respectively [5–9]. This study used astaxanthin (C_40_H_52_O_4_) as a natural sensitizer. Astaxanthin has maximum absorption in the visible range, between 474 and 478 nm. Astaxanthin consists of long conjugated hydrocarbon chains or a polyene system, which contains two cyclohexane ring systems at the terminal position of the structure. Hydroxyl (-OH) and carbonyl (C=O) groups are bound to both cyclohexane ring systems. The -OH and C=O groups of astaxanthin allow it to interact with the TiO_2_ surface through chelation to the Ti^4+^ site, thus allowing electrons injected into the conduction band of TiO_2_ [10].

The major challenge in developing DSSCs with natural dyes as sensitizers is their low stability caused by light and oxygen exposure. Efforts are needed to increase the stability of sensitizers, which can ultimately improve the efficiency of energy conversion and DSSC stabilities [11]. The stability improvement of astaxanthin or another carotenoid compound can be carried out by several methods, such as impregnation [12], encapsulation [13,14], complexation [15], and combining dye molecules with other materials [16]. The encapsulation and impregnation methods have drawbacks, such as the difficulty of controlling the particle size and the high temperatures during the synthesis process that could increase the degradation of natural dye [17].

In this research, astaxanthin was complexed with transition metal ion Zn^2+^. The choice of transition metal ions was based on the Lewis hard and soft acid bases (HSAB), where the ion Zn^2+^ belongs to the borderline acids group, which will fast bond with the atom O from the carbonyl group (C=O) of astaxanthin as a soft Lewis base [18]. This study also investigated the performance of DSSCs with astaxanthin and complex Zn-astaxanthin as sensitizers. The parameters determined are the open-circuit voltage (V_OC_), short-circuit current (I_SC_), and maximum energy conversion efficiency. The stability due to the irradiation process by halogen lamps was determined based on the maximum energy conversion efficiency produced by DSSC. The stability data of DSSC can be used as information on DSSC in the long-term application.

## 2. Materials and methods

### 2.1. Materials

(3S,3S′)-*trans*-astaxanthin (≥97%; Sigma Aldrich), NH_4_OH-activated metakaolinite (MKA) prepared by Rahmalia et al. [19]. Acetone (CH_3_COCH_3_, 99.8%, Mallinckrodt Chemicals), iodine (I_2_) by VWR Chemicals, acetonitrile (C_2_H_3_N, 99.8%), potassium iodide (KI), triton-x-100 (C_8_H_17_C_6_H_4_(OCH_2_)_n_OH), acetylacetone (C_5_H_8_O_2_), absolute ethanol (C_2_H_5_OH), aluminium thin layer chromatography plate, silica gel coated with fluorescent indicator F254 (TLC F_254_), 1-methyl-3-prophylimidazolium iodides (C_8_H_13_IN_2_), and zinc(II)sulfate hydrate (ZnSO_4_.H_2_O) were supplied by Sigma Aldrich. Plastisol was supplied by Solaronix; transparent conductive oxide (TCO) glass with fluorine-doped tin oxide (FTO) type thermal evaporation coating, 7–8 ohm/seq (TEC-7) conductive glass by SOLEM.

### 2.2. Methods

#### 2.2.1 Preparation of complexes Zn-astaxanthin

The Zn-astaxanthin complex was synthesized by adopting the method of Rahmalia et al. [15]. A metal precursor, ZnSO_4_.H_2_O, was heated at 100 °C for 24 h to remove the hydrate. A Zn^2+^ aqueous solution (14.7 mg in 1 mL of distilled water) was added to the astaxanthin solution (54.5 mg in 30 mL of acetone) with a molar ratio of 1/1. The mixture was stirred with a magnetic stirrer until homogeneous. Reflux was carried out at 37 °C for 1 h and continued at 60 °C for 1 h. The resulting product (Zn-astaxanthin) was then analyzed using ^1^H-NMR spectroscopy.

#### 2.2.2 Fabrication of dye-sensitized solar cell (DSSC)

##### Preparation of transparent conductive oxide (TCO)

TEC7 FTO glass measuring 2 × 2 cm^2^ was washed using soap and water, then the glasses were immersed in 70% ethanol and sonicated for 30 min. After that, the glasses were dried at 100 °C for 1 h, and the conductive side of the glasses was determined using a multimeter.

##### Preparation of photoelectrode

Photoelectrode was prepared by adopting the method of Rahmalia et al. [19]. The photoelectrode used was a mixture of TiO_2_ anatase (2.3751 g), NH_4_OH-activated metakaolinite (MKA) (0.1264 g), 15 mL of absolute ethanol, 16 drops of triton-x-100, and 12 drops of acetylacetone. The mixture was stirred for 24 h to form a paste, followed by sonication for 3 h.

##### Preparation of electrolyte

Electrolyte was prepared by adopting the method of Rahmalia et al. [5] and Rahmalia et al. [15]. The 0.05 M I_2_ was added to the KI solution (1.66 g in 20 mL of acetonitrile). The mixture was stirred until homogeneous. An amount of 5 mL of 1-methyl-3-prophylimidazolium iodide (MPII) 0.4 M ionic liquid was added to the mixture and was shaken carefully. The electrolyte solution was stored in a dark and closed bottle.

##### Assembling of DSSCs

Two pieces of TCO glass with an active side of 1 × 1 cm^2^ were prepared. Photoelectrode paste was dripped onto the surface of one glass using the doctor blade method. The glass was then heated in a furnace at a temperature of 450 °C for 30 min. Meanwhile, platinum paste was dripped into the other glass (as a counter electrode) and heated at a temperature of 400 °C for 5 min.

**Figure 1:**
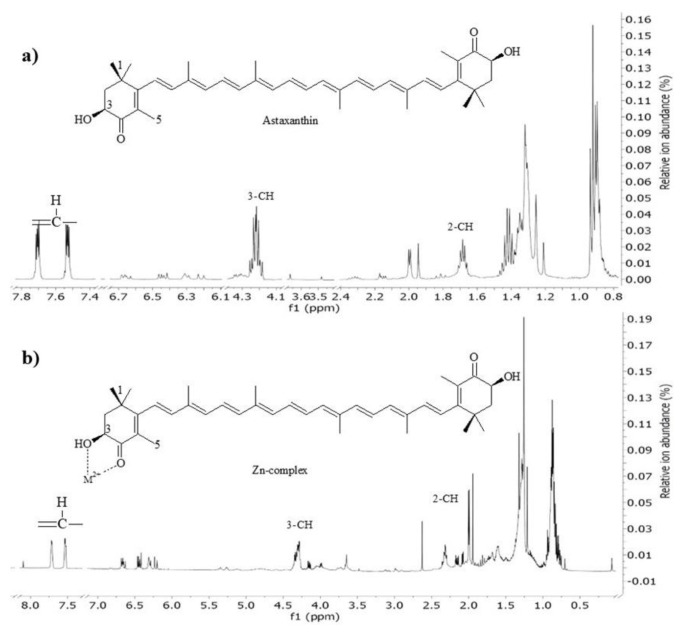
^1^H-NMR (500 MHz) spectra of astaxanthin (a) and Zn-astaxanthin (b).

Photoelectrode was immersed in a sensitizer solution (36 g/L of Zn-astaxanthin in acetone) for 24 h. Furthermore, one drop of electrolyte was added and then covered with FTO glass which has been coated with a counter electrode to form a layer like a sandwich (Figure 1). As a comparison, DSSC with pure astaxanthin as a sensitizer was made using the same method. DSSCs using Zn-astaxanthin and astaxanthin were made in 3 cells each for repetition of the test in triplicate.

In the discussion section, photoelectrode immersed in Zn-astaxanthin and pure astaxanthin are called PE/Zn-astaxanthin and PE/astaxanthin respectively. In addition, the DSSC using Zn-astaxanthin and astaxanthin as sensitizers are called DSSC/Zn-astaxanthin and DSSC/astaxanthin, respectively.

The performance test was carried out by illuminating the DSSCs using a 500-watt halogen lamp with a light intensity of 0–1000 W/m^2^. The light intensities were measured using a solar power meter. The short-circuit current (I_SC_) and open-circuit voltage (V_OC_) were measured using a multimeter 6.5 digits PICOTEST M3510. Furthermore, the stability of the cells was tested by illuminating the cells at maximum intensity for 10 h, determining the I_SC_ and V_OC_ values of the cells with an illumination period of 1 h. Cells were rested for 14 h in the dark and reilluminated continuously. Measurements were carried out for a total time of 70 h.

## 3. Results and discussion

### 3.1. ^1^H-NMR measurements

The complex formation of astaxanthin with the transition metal ion (Zn^2+^) was observed using ^1^H-NMR (500 MHz) with chloroform as a solvent. The ^1^H-NMR spectra of Zn-astaxanthin compared to pure astaxanthin are shown in Figure 1. The maximum change in the chemical shift was observed for the 3-CH proton nearest to the -OH group, another proton of the cyclohexane ring, and the 2-CH proton these changes are +0.1, 7.52–7.73, and +0.64 ppm respectively (Table).

### 3.2. Interaction between sensitizer with photoelectrodes

The semiconductor used in this research was TiO_2_ anatase. Anatase crystal form has shown a more stable catalytic performance than the rutile phase, with a slow electron recombination process [20]. Park [21] reported that anatase TiO_2_ has a higher incident photon to current conversion efficiency (IPCE) value than rutile TiO_2_. Our previous research reported the adsorption of bixin as a sensitizer on the TiO_2_ anatase surface followed the model of Langmuir (chemistry interaction), and the performance test of DSSC showed higher energy conversion efficiency than commercial TiO_2_ P-25 [19,22].

**Figure 2:**
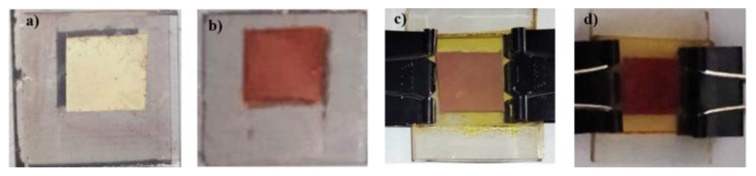
PE/astaxanthin (a), PE/Zn-astaxanthin (b), DSSC/astaxanthin (c), DSSC/Zn-astaxanthin (d).

**Figure 3:**
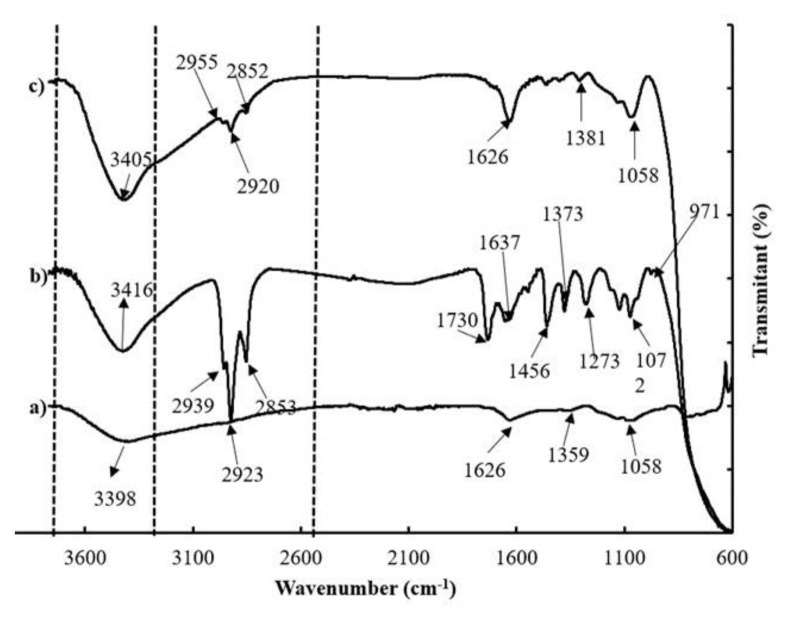
FT-IR spectra of PE (a), PE/Zn-astaxanthin (b), and PE/astaxanthin (c).

**Figure 4:**
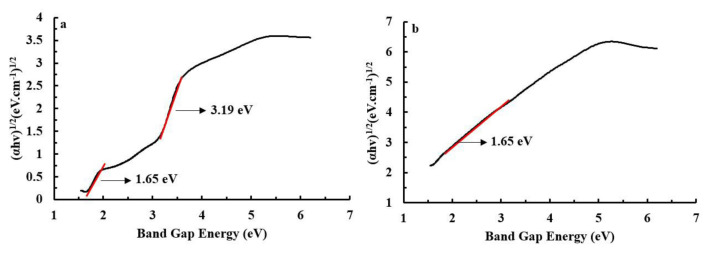
DRS UV-Vis result based on Tauc plot method for PE/astaxanthin (a) and PE/Zn-astaxanthin (b).

Based on Figure 2, it can be seen that there is a color difference on the TiO_2_ surface after being immersed in a solution of astaxanthin (PE/astaxanthin) and Zn-astaxanthin complex (PE/Zn-astaxanthin) sensitizer for 24 h. The PE/Zn-astaxanthin has a deeper color. This is probably due to a better interaction between the Zn-astaxanthin complex and the TiO_2_ surface compared to pure astaxanthin as a sensitizer. The interaction between the TiO_2_ surface and the sensitizer was then observed based on the results of FTIR (Figure 3) and UV-Vis DRS (Figure 4) analysis.

The FTIR spectrum for the photoelectrode used in this study (Figure 3a), shows the existence of stretching vibrations for the -OH functional group (from the water group) at 3398 cm^−1^, -OH deformation stretching vibrations at 1626 cm^−1^; stretching vibration of Ti-O-Ti and O-Ti-O at 1359 cm^−1^, and stretching vibration of Si-O (Si-O-Si and O-Si-O) at 1058 cm^−1^. The FTIR results are in accordance with research conducted by Rahmalia et al. [15]. After being immersed in astaxanthin and Zn-astaxanthin sensitizers, the shift of -OH stretching vibrations peaks is observed, corresponding to both inter- and intramolecular hydrogen bonds. The change of inter- and intramolecular hydrogen bonds may be due to the interaction between the PE surface with -OH groups of the sensitizers [23].

Furthermore, in the spectrum of the PE/Zn-astaxanthin (Figure 3b), stretching vibrations were observed for the carbonyl (C=O) and the hydroxyl (-OH) functional group at 1730 and 1273 cm^−1^ respectively, where the absorption peak was not observed on the FTIR spectrum of PE/astaxanthin. In the FTIR spectrum of the PE/Zn-astaxanthin, it can also be observed that there is absorption for the H-C-H stretching vibration at 2939, 2923, and 2853 cm^−1^ with a higher absorption intensity compared to the PE/astaxanthin, indicating that more Zn-astaxanthin sensitizer is bound on the photoelectrode compared with pure astaxanthin.

Determination of the photoelectrode band gap energy was carried out using the Tauch plot method. Rahmalia et al. [15] reported that the TiO_2_ semiconductor added with 5% NH_4_OH-activated metakaolinite as adopted in this study, had a band gap energy of 3.16 eV (equivalent to 393 nm). After the photoelectrode was soaked with a sensitizer for 24 h, there was a change in the band gap energy (Figure 4). PE/astaxanthin has two band gap energies, namely 3.19 and 1.65 eV (equivalent to 388 and 752 nm) (Figure 4a). Meanwhile, there is only one band gap energy of PE/Zn-astaxanthin, namely 1.59 eV (equivalent to 780 nm) (Figure 4b).

The significant decrease in photoelectrode band gap energy after immersion with a sensitizer was due to astaxanthin and Zn-astaxanthin originally having low band gap energies of 2.487 and 0.365 eV respectively [24]. Good adsorption of the sensitizer onto the surface of the TiO_2_ semiconductor will make a large contribution to lowering the band gap energy of TiO_2_. Therefore, in its application to DSSC systems, a small band gap energy will widen the light absorption area in the UV-Vis wavelength range and increase energy conversion efficiency.

### 3.3. DSSC performance and stability

The open-circuit voltage (V_OC_) and circuit current (I_SC_) are key parameters that govern the attainable power from a DSSC. The V_OC_ is the maximum voltage available from a DSSC that occurs at zero current, while I_SC_ is the current through the DSSC when the voltage across is zero. Maximum energy conversion efficiency is an energy conversion efficiency when the fill factor (FF) value is ignored [5].

**Figure 5:**
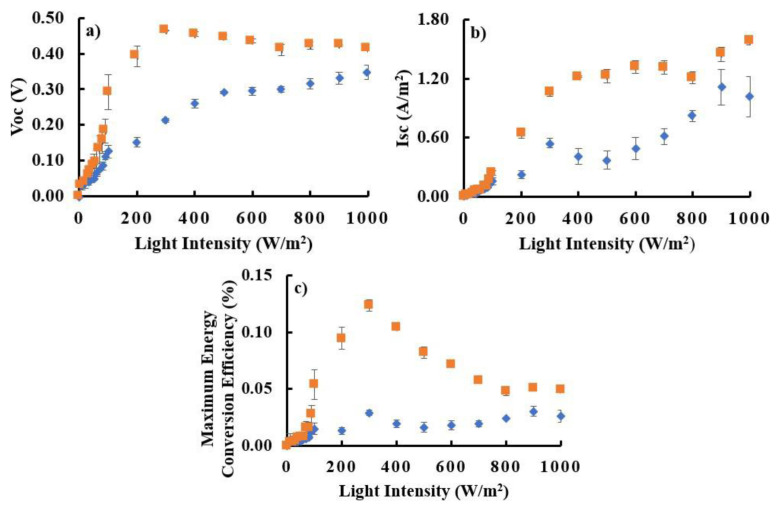
Performance parameters of DSSC/astaxanthin ( 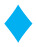) and DSSC/Zn-astaxanthin ( 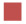): correlation between light intensity to V_OC_ (a), I_SC_ (b), and maximum energy conversion efficiency (c).

Figure 5 shows the relationship between light intensity and V_OC_ (a) and I_SC_ (b). Under illumination with an intensity of 0–300 W/m^2^, the V_OC_ and I_SC_ values for DSSC with astaxanthin and Zn-astaxanthin sensitizers (DDSC/Zn-astaxanthin and DSSC/astaxanthin) tend to increase significantly. Maximum energy conversion efficiency occurs when the DSSC is exposed to light with an intensity of 300 W/m^2^. Under these conditions, the energy conversion efficiency of DSSC/astaxanthin and DSSC/Zn-astaxanthin is (0.03 ± 0.0022)% and (0.12 ± 0.0052)%, respectively (Figure 5c). The maximum energy conversion efficiency decreases when the DSSC is illuminated with an intensity above 300 W/m^2^. This phenomenon occurs because the increase in intensity is accompanied by an increase in temperature, thereby reducing DSSC performance. The dramatic decrease in the maximum energy conversion efficiency value was caused by an increase in the light intensity value displayed on the DSSC.

The maximum energy conversion efficiency for DSSC/Zn-astaxanthin showed higher results than DSSC/astaxanthin, this corresponds to the band gap energy value of PE/Zn-astaxanthin which is much lower compared to PE/astaxanthin. A low band gap energy causes less energy needed to move electrons in the valence band to the conduction band of a semiconductor. Rahmalia et al. [15], reported that semiconductors with low band gap energy are responsible for increasing electron injection and transport to increase high energy conversion efficiency.

**Figure 6:**
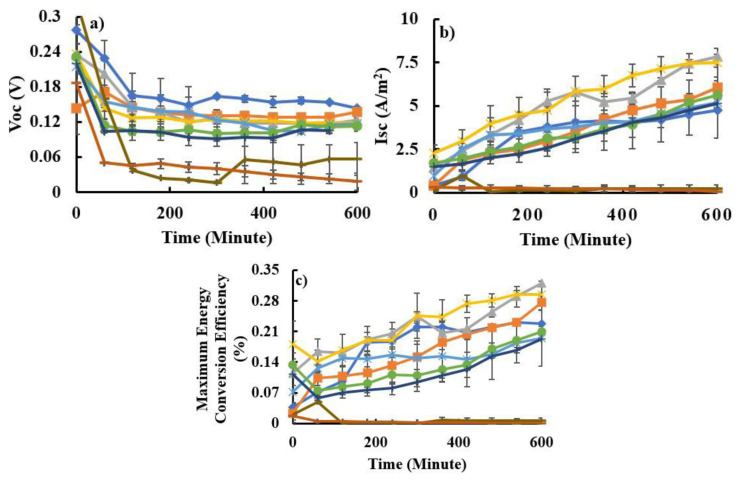
Stability and reusability of DSSC/astaxanthin (day-1 ( 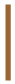), and day-2 ( 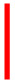)) and DSSC/Zn-astaxanthin (day-1 ( 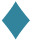), day-2 ( 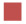), day-3 ( 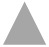), day-4 ( 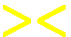), day-5 ( 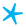), day-6 ( 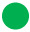), day-7 ( 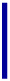)) based on V_OC_ (a), I_SC_ (b), and maximum energy conversion efficiency (c).

Stability tests were carried out by irradiating the DSSC at maximum light intensity (300 W/m^2^) for 10 h continuously. After the DSSC was rested in the dark for 14 h, the DSSC was reilluminated. The test results showed that DSSC/Zn-astaxanthin which was irradiated for 7 days (total irradiation time was 70 h) still showed relatively stable performance. In contrast, the DSSC/astaxanthin sensitizer only lasted for 2 days (total irradiation 20 h) (Figure 6), characterized by a drastic decrease in V_OC_ and I_SC_ values.

DSSC/Zn-astaxanthin has much better stability than DSSC/astaxanthin, proving that the complexation process of astaxanthin with transition metal ions (Zn^2+^) causes good interaction between the sensitizer and the TiO_2_ surface. This can stabilize DSSC performance under continuous light exposure and increasing temperature during the irradiation process. The high I_SC_ DSSC value when using the Zn-astaxanthin sensitizer is influenced by the ability to absorb photon energy better, because the transition metal ion Zn^2+^ has properties as a redox catalyst and only has one valence state, so at high temperatures during the cell irradiation process, Zn-astaxanthin was more stable than astaxanthin [25].

The phenomenon of decreasing V_OC_ values (voltage drop) in both DSSC/Zn-astaxanthin and DSSC/astaxanthin is the main indicator of power quality which occurs along with increasing impedance or electric current [26]. In this study, long-term irradiation also caused an increase in temperature from 32 °C (before irradiation) to 60 °C (after 10 h of irradiation), and the resistance also increased from 13.3699 to 58.7836 kΩ. However, decreasing the voltage value does not cause a decrease in the maximum energy conversion efficiency value of DSSC-Zn-Astaxanthin because the resulting current becomes higher as time increases. The increase in I_SC_ can be attributed to photoinduced annealing, then improving overall performance. This can occur, for example, by recovering and/or rearranging some of the dangling or taut bonds between the sensitizer and the photoelectrode or within the dye molecules [27].

## 4. Conclusions

The Zn-astaxanthin complex has been successfully synthesized in this research. The complexation method has been proven to increase the stability of astaxanthin so that it can improve DSSC performance when this complex compound is used as a sensitizer. DSSC using Zn-astaxanthin as a sensitizer showed a maximum energy conversion efficiency four times higher compared to DSSC using a pure astaxanthin sensitizer. DSSC which uses Zn-astaxanthin as a sensitizer still shows stable performance up to irradiation for a total of 70 h.

## Figures and Tables

**Table:** ^1^H-NMR (500 MHz) spectra of astaxanthin and Zn-astaxanthin complex.

	3-CH	2-CH_2_	=CH-
Astaxanthin	4.22	1.68	7.69–7.72
Zn-astaxanthin	4.32 (+0.10)	2.32 (+0.64)	7.52–7.73
